# Cataract Extraction After Brachytherapy for Malignant Melanoma of the Choroid in a Young Female Patient: A Case Report

**DOI:** 10.7759/cureus.78253

**Published:** 2025-01-30

**Authors:** Birkaran Sadhar, Paarth Sharma, Tayyab Shakoor, Satnam Singh, Chris Buzas

**Affiliations:** 1 Ophthalmology, Lake Erie College of Osteopathic Medicine, Erie, USA; 2 Emergency Medicine, Lake Erie College of Osteopathic Medicine, Erie, USA; 3 Medicine, Conemaugh Memorial Medical Center, Johnstown, USA

**Keywords:** cataract patients, choroidal malignant melanoma, malignant uveal melanoma, ocular brachytherapy, ocular radiation

## Abstract

The use of brachytherapy, a form of radiation therapy, plays a crucial role in the management of intraocular melanomas of the choroid. Ionization radiation treatment has the unfortunate side effect of developing cataracts prematurely. We present an uncommonly seen case of a 36-year-old woman who presented with blurred vision in her left eye following successful plaque radiotherapy four years prior for malignant melanoma in the same eye. The patient had developed a cataract, which hindered her visual acuity and visualization of the tumor. Prompt cataract surgery after brachytherapy for radiation-induced cataracts is necessary for the improvement of the quality of life and ensuring effective uveal and retinal examination.

## Introduction

Malignant melanoma of the choroid, the most common primary intraocular malignancy in adults, has a strong propensity for metastasis to the liver [1]. Brachytherapy has assumed an increasingly important role in the management of uveal melanomas owing to advances in radiation technology and an appreciation of the limitations of enucleation [2]. Brachytherapy with radiation seeds iodine-125 (I-125) or ruthenium-106 is reserved for malignant tumors of the eye that are less than 3.5 mm in height and 16 mm in the largest basal diameter [3].

Brachytherapy has been reported to cause cataracts in other ophthalmic conditions, the most common being retinoblastoma in children. The etiology of radiation-induced cataracts is still unknown but is thought to be caused by direct damage to lens epithelial cells and the subsequent loss of the proliferative capacity of these cells [1,4].

High doses of ionizing radiation have been reported to cause an acute onset of cataracts in as little as six months. However, low doses of ionizing radiation may not cause cataracts until as late as 10 years after exposure [5]. This is important to recognize so that patients with uveal melanoma who undergo brachytherapy are followed by an ophthalmologist for the possible development of a radiation-induced cataract. This is especially essential in young patients as they generally have high visual demands and a long life expectancy and are at a higher risk of complications related to radiation treatment in subsequent years [1,6].

Brachytherapy has become the most commonly used radiotherapy for the conservative treatment of uveal melanoma and has been found to be effective in controlling tumor growth with few complications. Cataract formation following brachytherapy for malignant melanoma of the choroid is uncommon in young subjects such as our patient [3,4,7-9].

The purpose of this case report is to contribute to the existing literature on brachytherapy-induced cataracts and to highlight the potential benefits of timely cataract surgery following brachytherapy in young patients with uveal melanoma.

## Case presentation

A 36-year-old Caucasian woman presented with blurred vision in her left eye and was referred for cataract evaluation. Four years prior, she was diagnosed with choroidal melanoma in the same eye and underwent successful plaque radiotherapy to halt tumor growth. She was referred by her retina specialist due to the limited visualization of her choroidal neoplasm.

**Figure 1:**
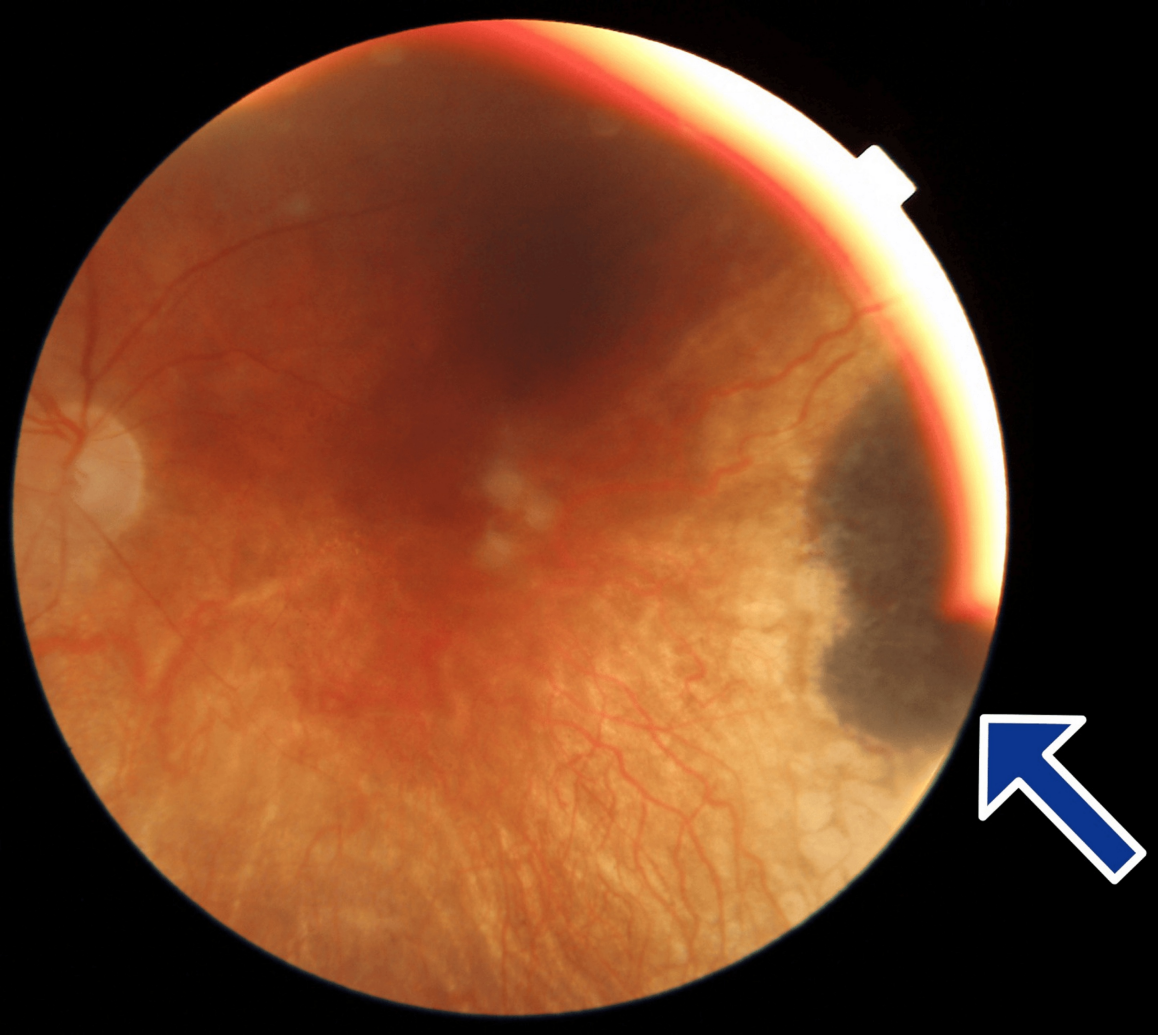
Fundus Imaging of the Choroidal Melanoma of the Left Eye

On examination, the best corrected visual acuity (BCVA) was 20/20 in the right eye/oculus dexter (OD) and hand motion (HM) in the left eye/oculus sinister (OS). Intraocular pressures (IOPs) were 20 and 16 mmHg in the OD and OS, respectively. The pupils were equal, round, and reactive, and extraocular movements were full. Anterior segment examination was unremarkable in the OD and revealed trace nuclear sclerotic with 4+ posterior subcapsular cataract in the OS. Posterior segment examination was unremarkable in the OD. Posterior segment examination in the OS revealed optic disc atrophy, temporal atrophy, fibrosis of the macula, and one hyperpigmented lesion 10 mm wide in the temporal peripheral retina, which has remained stable (Figure 1).

The patient was informed that upon cataract extraction and intraocular lens (IOL) implantation, visual acuity should improve but will be limited due to the treatment of the choroidal neoplasm and subtle optic disc atrophy. The procedure was discussed in detail, and the patient elected to undergo the surgery.

Cataract extraction was successfully performed with phacoemulsification with no complications, and a monofocal IOL was implanted. The patient was prescribed Ilevro (nepafenac) 0.3% eye drops once a day for four weeks, Vigamox (moxifloxacin) 0.5% eye drops three times a day for one week, and Durezol (difluprednate) 0.05% eye drops with tapering orders.

The patient was followed up on day 1, week 1, and month 1 after the operation. All evaluations involved her visual acuity, intraocular pressure (IOP), and the status and positioning of the intraocular lens.

On postoperative day 1, while the patient reported limited pain and her IOP was measured at 18 mmHg in the OS, her BCVA remained quite poor at 20/200 pinhole in the OS. Her intraocular lens was situated well. She was counseled to continue her postoperative drops as prescribed.

At the one-week follow-up, the patient affirmed medication adherence since discharge. Unfortunately, no improvement was seen in her vision, which remained at 20/200 pinhole in the OS. Her IOP in the OS had increased to 22 mmHg. It was determined wise to commence a taper of her steroid treatment.

By the one-month mark, signs of progress emerged. Her IOP decreased to a suitable 14 mmHg in the OS, and an improvement in vision was noted as 20/100 pinhole in the OS. Additionally, her choroidal melanoma appeared stationary under surveillance. She continues routine monitoring with her retinal specialist.

## Discussion

Treating choroidal melanoma in young patients requires long-term surveillance, the management of concurrent cataract formation from radiotherapy, and the consideration of the quality of life impacts. This unique situation highlights the importance of understanding the underlying pathophysiology and clinical and surgical management of choroidal melanoma treatment-induced cataracts in younger patients.

A significant aspect of this case is the presence of choroidal melanoma in an individual as young as 36, which is uncommon [3,4,7-9]. The utilization of brachytherapy as part of the treatment increased the intricacy of the management of this patient as it is effective in halting tumor growth but risks inducing cataract formation [1,5].

The rate of cataract formation in patients undergoing radiation therapy is highly variable as it is influenced by tumor location, increased patient age, tumor size, and radiation dose to the lens [9-11]. Tumors that are more anterior are closer to the lens and thus increase the amount of radiation exposure to the lens. One study highlighted that patients developed cataracts a median of 15 months sooner in cases of anterior tumors than posterior tumors and that a greater proportion of cataracts occurred in patients with anterior tumors [12]. Patients who are older may already have nuclear sclerotic cataracts forming, and brachytherapy has the potential to worsen existing nuclear sclerotic cataracts [13]. Larger tumors tend to have decreased time to cataract formation and an increased risk of cataract development, potentially due to exposure to a higher radiation dose [13]. The Collaborative Ocular Melanoma Group found a direct relationship between the cumulative dose of the first five years of I-125 brachytherapy and the incidence of cataract development. Cumulative doses of ≥24 Gy, 16-23.9 Gy, 12-15.9 Gy, and <12 Gy were associated with a 92%, 88%, 86%, and 65% cataract incidence, respectively [14].

In the present case, the radiation-associated cataract developed and progressed over a four-year period. Details regarding her brachytherapy dose were not available, and a 4+ posterior subcapsular cataract was noted in her left eye. The young age of our patient and the considerable effect of the cataract on her quality of life favored cataract extraction and IOL implantation. Given that there are no standard guidelines for the treatment of radiation-induced cataracts, the timing of cataract surgery must be individualized and weighed against the risk of cataract progression. In the case of our patient, earlier cataract surgery might have prevented a decrease in the quality of life in the patient due to her poor visual acuity in the OS. Furthermore, cataract extraction was necessary to monitor her choroidal neoplasm.

Several studies have explored potential strategies to mitigate the risk of post-radiotherapy complications. Shield et al. investigated the use of bevacizumab (Avastin) as a posttreatment intervention, suggesting its potential in preventing visual acuity complications from radiation retinopathy [5].

Thariat et al. investigated whether sparing the lens during proton therapy could lessen the risks of cataracts forming [15]. Using this method, researchers were able to avoid radiation to the lens in 25% of the cases. They also found that many patients demonstrated higher radiation tolerance than previously thought, remaining vision-impairing-cataract-free even when exposed to doses exceeding the standard 0.5 Gy threshold, provided the radiation was limited to only a portion of the lens [15]. This proposed proton therapy can be customized to minimize radiation exposure to the lens, potentially cutting the need for cataract operations while still effectively treating the tumor. Furthermore, minimizing the radiation dose delivered during plaque therapy has been suggested as a possible approach, although its feasibility may depend on specific tumor characteristics [9].

An earlier study indicated that verapamil might influence the development of radiation-induced cataracts. Rats were exposed to radiation, and the concentrations of various minerals in their lenses were determined. Researchers discovered that calcium levels were higher in the lenses of the radiation-treated group than in the control group and that verapamil significantly lowered the calcium lens concentration in the radiation-treated group. The study indicates that verapamil may prevent cataracts in these cases by reducing lens calcium levels, which is a point believed to be important in their formation [16]. Similar studies should be investigated in human subjects.

This case sheds new light as the affected individual is considerably younger than usual for developing cataracts after receiving brachytherapy to eradicate choroidal melanoma. The atypical features of our patient's clinical presentation and medical history broaden our understanding of how this treatment can sometimes precipitate lens opacity in those who are far from the typical profile. Such outlier cases expand our perspective on the interrelations between age, posterior uveal melanomas, plaque radiotherapy aimed at these tumors, and subsequent cataractogenesis.

While this case study offers valuable insights, additional studies are warranted to confirm the broader applicability of these observations. Moreover, further research should investigate methods to mitigate the formation of visually significant cataracts following brachytherapy.

## Conclusions

Ophthalmologists need to recognize that patients with uveal melanoma who undergo brachytherapy have a high likelihood of developing radiation-induced cataracts. Young patients, as seen in this case, often have high visual demands, and the prompt treatment of the cataract following brachytherapy shows promising results. Since there are no standard guidelines for the treatment of radiation-induced cataracts, treatment plans should be tailored to each individual patient.
